# Editorial: Machine learning-based methods for RNA data analysis—Volume II

**DOI:** 10.3389/fgene.2022.1010089

**Published:** 2022-11-29

**Authors:** Lihong Peng, Jialiang Yang, Minxian Wang, Liqian Zhou

**Affiliations:** ^1^ College of Life Sciences and Chemistry, Hunan University of Technology, Zhuzhou, China; ^2^ School of Computer, Hunan University of Technology, Zhuzhou, China; ^3^ Geneis (Beijing) Co Ltd., Beijing, China; ^4^ CAS Key Laboratory of Genome Sciences and Information, Beijing Institute of Genomics, Chinese Academy of Sciences, Beijing, China; ^5^ University of Chinese Academy of Sciences, Beijing, China

**Keywords:** machine learning, lncRNA, microRNA, circRNA, mRNA, gene expression

RNAs regulate multiple biological processes including RNA transcription, splicing, stability, and translation. They play significant roles in cell biology (Connelly et al. (2016); Licatalosi and Darnell (2010); Mukherjee et al. (2022); Chen et al. (2018b)). The Encyclopedia of DNA elements project reported that only 1.5% of human genome is translated into proteins, while approximately 70%–90% is transcribed to RNAs (Falese et al. (2021)). RNAs greatly expand the range of targets from proteins to RNAs by re-targeting mutated targets (Yu et al. (2019); Chen et al. (2020); Li et al. (2022); Yang et al. (2022)). Particularly, noncoding RNAs have dense linkages with human diseases including cancers. Now, RNAs have been diagnostic or prognostic markers of complex diseases (Hui et al. (2011); Xu et al. (2022); Peng et al. (2022a); Shen et al. (2022); Zhang T. et al. (2022); Chai et al. (2022)). In this topic, we aim to analyze diverse RNA data to provide clues for the diagnosis and therapy of various diseases (Dal Molin et al. (2022); Wang S. et al. (2022); Li J. et al. (2019); Liu et al. (2020)). Long noncoding RNAs (lncRNAs) regulate many significant biological processes (such as immune response and embryonic stem cell pluripotency) by linking to RNA-binding proteins (Wapinski and Chang (2011); Chen and Huang (2017); Ping et al. (2018); Wang et al. (2020)), Wang et al. (2021 W.); Peng et al. (2020)). They have been important biomarkers for cancers (Wu et al. (2022a); Banerjee et al. (2020); Zhang S. et al. (2021); Zhou G. et al. (2021); Peng et al. (2022a); Liang et al. (2022b); Peng et al. (2021); Zhou L. et al. (2021)). For example, lncRNAs AFAP1-AS1, CCAT1, CYTOR, GAS5, HOTAIR, and PVT1 are molecular regulators of lung caner (Aftabi et al. (2021)). KCNQ1OT1 may be a prognostic biomarker in colorectal cancer (Lin et al. (2021)). lncRNAs are also oncogenes (such as MKLN1-AS, GHET1, LASP1-AS, MALAT1, HULC, HOTAIR, and PAPAS) and tumor suppressors (such as CASC2, DGCR5, MEG3, GAS5, and NRON) in hepatocellular carcinoma (Guo et al. (2021)). Many machine learning methods have been proposed to infer new LncRNA-Disease Associations (LDAs). For example, graph convolutional completion with conditional random (Fan et al. (2022)), heterogeneous graph attention network with meta-paths (Zhao et al. (2022)), graph convolutional auto-encoders (Silva and Spinosa (2021)), multi-view attention graph convolutional network and stacking ensemble (Liang et al. (2022b)), and learning to rank-based model (Wu et al. (2022a)) are widely used methods for LDA prediction.

In this research topic, Sun et al. developed a weighted graph-regularized matrix factorization approach (LPI-WGRMF) to identify possible lncRNA-protein interactions (LPIs) based on known biological information and LPI matrix. LPI-WGRMF obtained an AUC of 0.9012 and AUPR of 0.7324 on LPI dataset provided by Zhang et al. (Zhang et al. (2018)) based on 5-fold cross validation. They predicted that lncRNAs SNHG3, SFPQ, and PRPF31 may interact with proteins Q9NUL5, Q9NUL5, and Q9UKV8, respectively. Yao et al. designed a random walk with restart algorithm (MHRWRLDA) to infer LDAs on multiplex and heterogeneous networks. MHRWRLDA computed an AUC of 0.6874 under leave-one-out cross validation, and inferred that lncRNA BCYRN1 may associate with colon cancer and hepatocellular carcinoma. Cheng et al. considered that the recurrence rate of nonfunctioning pituitary adenoma is relatively high after surgical resection and built lncRNA signatures for its prognosis. They obtained microarray sequencing profiles of lncRNA expressions from 66 patients who suffered from nonfunctioning pituitary adenoma. Univariable Cox regression analysis and random survival forests-variable hunting were applied to filter lncRNAs. They found that three lncRNAs, LOC101927765, RP11-23N2.4, and RP4-533D7.4, have dense associations with tumor recurrence and inferred that the three lncRNAs may be potential therapeutic targets of nonfunctioning pituitary adenoma.

MicroRNAs (miRNAs) are a class of endogenous noncoding RNAs with a length of approximately 22 nucleotides (Sun et al. (2022); Chen et al. (2019b, 2018b); Zhang L. et al. (2021)). MiRNAs regulate many biological activities and influence almost all genetic pathways (Chen et al. (2018c); Peng et al. (2017); Chen et al. (2018a)). Thus, miRNAs have been a class of tumor suppressor genes in clinical medicine (Chen et al. (2019a); Peng et al. (2018)). For example, miR-940 is a potential biomarker of prostate cancer (Rajendiran et al. (2021)). Urinary exosome microRNA signatures are noninvasive prognostic markers for prostate cancer (Shin et al. (2021)). Recently, machine learning methods have been widely used to identify possible MicroRNA-Disease Associations (MDAs). For example, tensor decomposition with relational constraints (Huang et al. (2021)), similarity constrained matrix factorization (Li L. et al. (2021)), tensor factorization and label propagation (Yu et al. (2022)), deep attributed network embedding model (Ji et al. (2021)), and multi-view multichannel attention graph convolutional network (Tang et al. (2021)) are popular methods in MDA prediction.

In this topic, Qu et al. explored a computational model (BRWRMHMDA) for MDA inference combining enforcing degree-based biased random walk with restart. BRWRMHMDA computed an AUC of 0.8310 under leave-one-out cross validation. They predicted that hsa-let-7f and hsa-mir-30e may associate with esophageal neoplasms and breast neoplasms, respectively. Zhou et al. proposed a pseudogene-miRNA association identification method (PMGAE) by integrating feature fusion, graph autoencoder, and eXtreme gradient boosting. First, they computed three types of similarities for pseudogenes and miRNAs, that is, Pearson similarity, cosine similarity, and Jaccard similarity. Second, the above similarities were fused to build a similarity profile for each node. Third, the similarity profiles and pseudogene-miRNA associations are further aggregated to depict each node as a low-dimensional vector through a graph autoencoder. Finally, the feature vector was fed into eXtreme gradient boosting for pseudogene-miRNA association prediction. PMGAE computed better AUC of 0.8634 and AUPR of 0.8966. The results from PMGAE showed that miRNAs hsa-miR-34c-5p, hsa-miR-199b-5p, and hsa-miR-103a-3p may associate with pseudogenes RPLP0P2, HLA-H, and HLA-J, respectively.

Circle RNAs (circRNAs) is a class of novel endogenous noncoding RNAs with a covalently closed loop structure (Wang C.-C. et al. (2021); Li G. et al. (2019); Wang et al. (2021b)). circRNAs have more stable expressions due to their resistances to RNA exonuclease degradation (Li et al. (2020); Wang et al. (2021c,b)). They can regulate protein binding, miRNA sponges, alternative splicing and transcription, and generate pseudogenes (Wang C.-C. et al. (2021); Chen (2020)). In addition, they demonstrate close associations with cancers, cardiovascular and nervous system diseases (Wang C.-C. et al. (2021); Li G. et al. (2019, 2020); Wang et al. (2021c,c,b)). Therefore, various computational models have been developed to detect possible CircRNA-Disease Associations (CDAs). For example, network embedding and subspace learning method (Xiao et al. (2021)), knowledge attention network (Lan et al. (2022)), multi-source feature fusion-based machine learning framework (Wang L. et al. (2022)), and robust nonnegative matrix factorization model (Peng et al. (2022c)) are widely used in CDA prediction.

Furthermore, Li et al. developed a computational CDA identification method (GATGCN) based on graph attention network and graph convolutional network. First, they fused several biomedical data from different sources through the centered kernel alignment model. Second, graph attention network was deployed to obtain latent representation of circRNAs and diseases. Finally, graph convolutional network was explored to infer CDAs. GATGCN computed better an AUC of 0.951 under leave-one-out cross validation and an AUC of 0.932 under 5-fold cross-validation. They found that circRNAs hsa_circRNA_404833, hsa_circ_0013509, hsa_circRNA_2149, circR_284, and circR_284 have the highest association scores with lung cancer, diabetes retinopathy, prostate cancer, cholangiocarcinoma, and clear cell renal cell carcinoma, respectively.

A large quantity of transcriptomic data enable us to investigate complex biological processes at single-cell resolution levels (Peng et al. (2022b); Liang et al. (2022a); Zhang et al. (2022b); Xu et al. (2020). Therefore, Miao et al. (2021) considered specific noises and computing efficiency, and then designed biologically interpretable integration strategies to integrate multi-omics single-cell data. Zhou P. et al. (2021) used multiscale stochastic dynamics to dissect transition cells from transcriptome data. Ye et al. (2022) used combinatorial hybrid sequencing to construct the axolotl cell landscape at single-cell resolution. McKellar et al. (2021) detected transitional progenitor states in mouse skeletal muscle regeneration based on single-cell transcriptomic data. Wu et al. (2022b) exploited a stacking ensemble learning-based model to implement single-cell Hi-C classification.

In particular, Panchy et al. analyzed large-scale transcriptome datasets using non-negative principal component analysis and non-negative matrix factorization. The results showed that the above two methods provided low-dimensional features for the progression of biological processes. They found that gene expression signatures from conserved epithelial-mesenchymal transition can be applied to depict the stages in multiple cell lines. Lang et al. evaluated the performance of two sequencing platforms (Nextseq500 and MGISEQ-2000) using the same capture DNA libraries built by the Illumina protocol. The results demonstrated that a significant loss of fragment occurred in the range of 101–133 bp sizes on MGISEQ-2000 for Illumina libraries while not for the capture DNA libraries. Bao et al. considered that it is crucial to differentiate the transcriptomic and proteomic profiles between unstable and stable atherosclerotic plaques. They obtained 5 unstable and 5 stable human carotid atherosclerotic plaques by carotid endarterectomy to identify lncRNA-targeted genes and circRNA-originated genes. The results indicated that 293 proteins, 488 lncRNAs, 91 circRNAs, and 202 mRNAs are differentially expressed between unstable and stable atherosclerotic plaques. Furthermore, CD5L, S100A12, CKB, CEMIP, and SH3GLB1 may be key genes in regulating the stability of atherosclerotic plaques. In addition, Zheng et al. used a series matrix file search method and obtained data related to breast cancer from the ArrayExpress and Gene Expression Omnibus databases. They found that RSK2 is a possible biomarker in breast cancer.

RNA sequencing data have been broadly applied to screen therapeutic strategies for various diseases (Przybyla and Gilbert (2022); Zhang Y. et al. (2021); Li C.-x. et al. (2021)). Chen et al. (2022) used RNA sequencing to explore the mechanism of oxygen-boosted sonodynamic therapy for the treatment of hepatocellular carcinoma. Zhang et al. (2022c) integrated single-cell and bulk RNA sequencing data to probe a pan-cancer stemness signature. Sammut et al. (2022) combined multi-omics data including DNA and RNA sequencing and machine learning technique to predict breast cancer therapy response. Based on RAN sequencing data, Ma et al. first downloaded RNA sequencing data related to gliomas from the TCGA database. Then they used DESeq2, key driver and weighted gene correlation network to identify differentially expressed genes. They observed that Paclitaxel, Cidofovir, 6-benzyladenine, Erlotinib, Bilirubin, Oxaliplatin, Nutlins, Valproic acid, and Fenofibrate may be potential drugs in inhibiting the recurrence of gliomas. Similarly, Xiang et al. detected gene expression and network differences between limited and advanced stages for the diffuse large B-cell lymphoma (DLBCL) patients to predict potential agents against DLBCL. First, they collected RNA sequencing data from the DLBCL patients at different clinical stages from the TCGA database. Second, they used DESeq2 to identify differentially expressed genes and weighted gene correlation network and differential modules to analyze variations between different stages. Finally, they extracted important genes using key drivers and identified potential agents for DLBCL patients using gene-expression perturbations and the CREEDS database. The results indicated that the thistle1 module had high association with the clinical stage of DLBCL. In addition, MOCOS, RAB6C, ACCSL, MMP1, and RGS21 were highly linked to the occurrence and development of DLBCL.

RNAs are a carrier of genetic information and have broad roles in regulating gene expression and other biological processes. Furthermore, the majority of noncoding RNAs are highly associated with diseases including cancers and nontumorigenic diseases. Thus, RNA data analysis contributes to prioritizing previously unrecognized therapeutic targets. We anticipate that this topic can provide clues for the diagnose and prognosis of complex diseases especially cancers.

## References

[B1] Aftabi Y. Ansarin K. Shanehbandi D. Khalili M. Seyedrezazadeh E. Rahbarnia L. (2021). Long non-coding rnas as potential biomarkers in the prognosis and diagnosis of lung cancer: A review and target analysis. IUBMB life 73, 307–327. 10.1002/iub.2430 33369006

[B2] Banerjee S. Yabalooru S. R. K. Karunagaran D. (2020). Identification of mrna and non-coding rna hubs using network analysis in organ tropism regulated triple negative breast cancer metastasis. Comput. Biol. Med. 127, 104076. 10.1016/j.compbiomed.2020.104076 33126129

[B3] Chai B. Ma Z. Wang X. Xu L. Li Y. (2022). Functions of non-coding rnas in regulating cancer drug targets. Acta Biochim. Biophys. Sin. 54, 279–291. 10.3724/abbs.2022006 35538038 PMC9828326

[B4] Chen L.-L. (2020). The expanding regulatory mechanisms and cellular functions of circular rnas. Nat. Rev. Mol. Cell. Biol. 21, 475–490. 10.1038/s41580-020-0243-y 32366901

[B5] Chen X. Guan N.-N. Sun Y.-Z. Li J.-Q. Qu J. (2020). Microrna-small molecule association identification: From experimental results to computational models. Briefings Bioinforma. 21, 47–61. 10.1093/bib/bby098 30325405

[B6] Chen X. Huang L. (2017). Lrsslmda: Laplacian regularized sparse subspace learning for mirna-disease association prediction. PLoS Comput. Biol. 13, e1005912. 10.1371/journal.pcbi.1005912 29253885 PMC5749861

[B7] Chen X. Wang L. Qu J. Guan N.-N. Li J.-Q. (2018a). Predicting mirna–disease association based on inductive matrix completion. Bioinformatics 34, 4256–4265. 10.1093/bioinformatics/bty503 29939227

[B8] Chen X. Xie D. Wang L. Zhao Q. You Z.-H. Liu H. (2018b). Bnpmda: Bipartite network projection for mirna–disease association prediction. Bioinformatics 34, 3178–3186. 10.1093/bioinformatics/bty333 29701758

[B9] Chen X. Xie D. Zhao Q. You Z.-H. (2019a). Micrornas and complex diseases: From experimental results to computational models. Brief. Bioinform. 20, 515–539. 10.1093/bib/bbx130 29045685

[B10] Chen X. Yin J. Qu J. Huang L. (2018c). Mdhgi: Matrix decomposition and heterogeneous graph inference for mirna-disease association prediction. PLoS Comput. Biol. 14, e1006418. 10.1371/journal.pcbi.1006418 30142158 PMC6126877

[B11] Chen X. Zhu C.-C. Yin J. (2019b). Ensemble of decision tree reveals potential mirna-disease associations. PLoS Comput. Biol. 15, e1007209. 10.1371/journal.pcbi.1007209 31329575 PMC6675125

[B12] Chen Y. Shang H. Wang C. Zeng J. Zhang S. Wu B. (2022). Rna-seq explores the mechanism of oxygen-boosted sonodynamic therapy based on all-in-one nanobubbles to enhance ferroptosis for the treatment of hcc. Int. J. Nanomedicine 17, 105–123. 10.2147/IJN.S343361 35027829 PMC8752973

[B13] Connelly C. M. Moon M. H. Schneekloth J. S. Jr (2016). The emerging role of rna as a therapeutic target for small molecules. Cell. Chem. Biol. 23, 1077–1090. 10.1016/j.chembiol.2016.05.021 27593111 PMC5064864

[B14] Dal Molin A. Gaffo E. Difilippo V. Buratin A. Tretti Parenzan C. Bresolin S. (2022). Craft: A bioinformatics software for custom prediction of circular rna functions. Brief. Bioinform. 23, bbab601. 10.1093/bib/bbab601 35106564 PMC8921651

[B15] Falese J. P. Donlic A. Hargrove A. E. (2021). Targeting rna with small molecules: From fundamental principles towards the clinic. Chem. Soc. Rev. 50, 2224–2243. 10.1039/d0cs01261k 33458725 PMC8018613

[B16] Fan Y. Chen M. Pan X. (2022). Gcrflda: Scoring lncrna-disease associations using graph convolution matrix completion with conditional random field. Brief. Bioinform. 23, bbab361. 10.1093/bib/bbab361 34486019

[B17] Guo C. Zhou S. Yi W. Yang P. Li O. Liu J. (2021). Long non-coding rna muskelin 1 antisense rna (mkln1-as) is a potential diagnostic and prognostic biomarker and therapeutic target for hepatocellular carcinoma. Exp. Mol. Pathol. 120, 104638. 10.1016/j.yexmp.2021.104638 33878313

[B18] Huang F. Yue X. Xiong Z. Yu Z. Liu S. Zhang W. (2021). Tensor decomposition with relational constraints for predicting multiple types of microrna-disease associations. Brief. Bioinform. 22, bbaa140. 10.1093/bib/bbaa140 32725161

[B19] Hui A. How C. Ito E. Liu F.-F. (2011). Micro-rnas as diagnostic or prognostic markers in human epithelial malignancies. BMC cancer 11, 500–509. 10.1186/1471-2407-11-500 22128797 PMC3260334

[B20] Ji B.-Y. You Z.-H. Wang Y. Li Z.-W. Wong L. (2021). Dane-mda: Predicting microrna-disease associations via deep attributed network embedding. Iscience 24, 102455. 10.1016/j.isci.2021.102455 34041455 PMC8141887

[B21] Lan W. Dong Y. Chen Q. Zheng R. Liu J. Pan Y. (2022). Kgancda: Predicting circrna-disease associations based on knowledge graph attention network. Brief. Bioinform. 23, bbab494. 10.1093/bib/bbab494 34864877

[B22] Li C.-x. Chen J. Lv S.-k. Li J.-h. Li L.-l. Hu X. (2021a). Whole-transcriptome rna sequencing reveals significant differentially expressed mrnas, mirnas, and lncrnas and related regulating biological pathways in the peripheral blood of Covid-19 patients. Mediat. Inflamm. 2021, 6635925. 10.1155/2021/6635925 PMC8018221 33833618

[B23] Li G. Luo J. Wang D. Liang C. Xiao Q. Ding P. (2020). Potential circrna-disease association prediction using deepwalk and network consistency projection. J. Biomed. Inf. 112, 103624. 10.1016/j.jbi.2020.103624 33217543

[B24] Li G. Yue Y. Liang C. Xiao Q. Ding P. Luo J. (2019a). Ncpcda: Network consistency projection for circrna–disease association prediction. RSC Adv. 9, 33222–33228. 10.1039/c9ra06133a 35529153 PMC9073279

[B25] Li J. Zhao H. Xuan Z. Yu J. Feng X. Liao B. (2019b). A novel approach for potential human lncrna-disease association prediction based on local random walk. IEEE/ACM Trans. Comput. Biol. Bioinform. 18, 1049–1059. 10.1109/TCBB.2019.2934958 31425046

[B26] Li L. Gao Z. Wang Y.-T. Zhang M.-W. Ni J.-C. Zheng C.-H. (2021b). Scmfmda: Predicting microrna-disease associations based on similarity constrained matrix factorization. PLoS Comput. Biol. 17, e1009165. 10.1371/journal.pcbi.1009165 34252084 PMC8345837

[B27] Li Y. Liang W. Peng L. Zhang D. Yang C. Li K.-C. (2022). Predicting drug-target interactions via dual-stream graph neural network. IEEE/ACM Trans. Comput. Biol. Bioinform. 2022, 1–11. 10.1109/TCBB.2022.3204188 36074878

[B28] Liang Y. Wu Y. Zhang Z. Liu N. Peng J. Tang J. (2022a). Hyb4mc: A hybrid dna2vec-based model for dna n4-methylcytosine sites prediction. BMC Bioinforma. 23, 258. 10.1186/s12859-022-04789-6 PMC9241225 35768759

[B29] Liang Y. Zhang Z.-Q. Liu N.-N. Wu Y.-N. Gu C.-L. Wang Y.-L. (2022b). Magcnse: Predicting lncrna-disease associations using multi-view attention graph convolutional network and stacking ensemble model. BMC Bioinforma. 23, 189. 10.1186/s12859-022-04715-w PMC9118755 35590258

[B30] Licatalosi D. D. Darnell R. B. (2010). Rna processing and its regulation: Global insights into biological networks. Nat. Rev. Genet. 11, 75–87. 10.1038/nrg2673 20019688 PMC3229837

[B31] Lin Z.-b. Long P. Zhao Z. Zhang Y.-r. Chu X.-d. Zhao X.-x. (2021). Long noncoding rna kcnq1ot1 is a prognostic biomarker and mediates cd8+ t cell exhaustion by regulating cd155 expression in colorectal cancer. Int. J. Biol. Sci. 17, 1757–1768. 10.7150/ijbs.59001 33994860 PMC8120463

[B32] Liu C. Wei D. Xiang J. Ren F. Huang L. Lang J. (2020). An improved anticancer drug-response prediction based on an ensemble method integrating matrix completion and ridge regression. Mol. Ther. Nucleic Acids 21, 676–686. 10.1016/j.omtn.2020.07.003 32759058 PMC7403773

[B33] McKellar D. W. Walter L. D. Song L. T. Mantri M. Wang M. F. De Vlaminck I. (2021). Large-scale integration of single-cell transcriptomic data captures transitional progenitor states in mouse skeletal muscle regeneration. Commun. Biol. 4, 1280. 10.1038/s42003-021-02810-x 34773081 PMC8589952

[B34] Miao Z. Humphreys B. D. McMahon A. P. Kim J. (2021). Multi-omics integration in the age of million single-cell data. Nat. Rev. Nephrol. 17, 710–724. 10.1038/s41581-021-00463-x 34417589 PMC9191639

[B35] Mukherjee D. Maiti S. Gouda P. K. Sharma R. Roy P. Bhattacharyya D. (2022). Rnabpdb: Molecular modeling of rna structure—From base pair analysis in crystals to structure prediction. Interdiscip. Sci. 14, 759–774. 10.1007/s12539-022-00528-w 35705797

[B36] Peng L.-H. Sun C.-N. Guan N.-N. Li J.-Q. Chen X. (2018). Hnmda: Heterogeneous network-based mirna–disease association prediction. Mol. Genet. Genomics 293, 983–995. 10.1007/s00438-018-1438-1 29687157

[B37] Peng L. Chen Y. Ma N. Chen X. (2017). Narrmda: Negative-aware and rating-based recommendation algorithm for mirna–disease association prediction. Mol. Biosyst. 13, 2650–2659. 10.1039/c7mb00499k 29053164

[B38] Peng L. Liu F. Yang J. Liu X. Meng Y. Deng X. (2020). Probing lncrna–protein interactions: Data repositories, models, and algorithms. Front. Genet. 10, 1346. 10.3389/fgene.2019.01346 32082358 PMC7005249

[B39] Peng L. Tan J. Tian X. Zhou L. (2022a). Enanndeep: An ensemble-based lncrna–protein interaction prediction framework with adaptive k-nearest neighbor classifier and deep models. Interdiscip. Sci. 14, 209–232. 10.1007/s12539-021-00483-y 35006529

[B40] Peng L. Wang C. Tian X. Zhou L. Li K. (2021). Finding lncrna-protein interactions based on deep learning with dual-net neural architecture. IEEE/ACM Trans. Comput. Biol. Bioinform. 2021, 3116232. 10.1109/TCBB.2021.3116232 34587091

[B41] Peng L. Wang F. Wang Z. Tan J. Huang L. Tian X. (2022b). Cell–cell communication inference and analysis in the tumour microenvironments from single-cell transcriptomics: Data resources and computational strategies. Brief. Bioinform. 23, bbac234. 10.1093/bib/bbac234 35753695

[B42] Peng L. Yang C. Huang L. Chen X. Fu X. Liu W. (2022c). Rnmflp: Predicting circrna–disease associations based on robust nonnegative matrix factorization and label propagation. Brief. Bioinform. 23, bbac155. 10.1093/bib/bbac155 35534179

[B43] Ping P. Wang L. Kuang L. Ye S. Iqbal M. F. B. Pei T. (2018). A novel method for lncrna-disease association prediction based on an lncrna-disease association network. IEEE/ACM Trans. Comput. Biol. Bioinform. 16, 688–693. 10.1109/TCBB.2018.2827373 29993639

[B44] Przybyla L. Gilbert L. A. (2022). A new era in functional genomics screens. Nat. Rev. Genet. 23, 89–103. 10.1038/s41576-021-00409-w 34545248

[B45] Rajendiran S. Maji S. Haddad A. Lotan Y. Nandy R. R. Vishwanatha J. K. (2021). Microrna-940 as a potential serum biomarker for prostate cancer. Front. Oncol. 11, 628094. 10.3389/fonc.2021.628094 33816263 PMC8017318

[B46] Sammut S.-J. Crispin-Ortuzar M. Chin S.-F. Provenzano E. Bardwell H. A. Ma W. (2022). Multi-omic machine learning predictor of breast cancer therapy response. Nature 601, 623–629. 10.1038/s41586-021-04278-5 34875674 PMC8791834

[B47] Shen L. Liu F. Huang L. Liu G. Zhou L. Peng L. (2022). Vda-rwlrls: An anti-sars-cov-2 drug prioritizing framework combining an unbalanced bi-random walk and laplacian regularized least squares. Comput. Biol. Med. 140, 105119. 10.1016/j.compbiomed.2021.105119 PMC8664497 34902608

[B48] Shin S. Park Y. H. Jung S.-H. Jang S.-H. Kim M. Y. Lee J. Y. (2021). Urinary exosome microrna signatures as a noninvasive prognostic biomarker for prostate cancer. NPJ Genom. Med. 6, 45–46. 10.1038/s41525-021-00212-w 34117264 PMC8196022

[B49] Silva A. B. O. V. Spinosa E. J. (2021). Graph convolutional auto-encoders for predicting novel lncrna-disease associations. IEEE/ACM Trans. Comput. Biol. Bioinform. 19, 2264–2271. 10.1109/TCBB.2021.3070910 33819159

[B50] Sun F. Sun J. Zhao Q. (2022). A deep learning method for predicting metabolite–disease associations via graph neural network. Brief. Bioinform. 23, bbac266. 10.1093/bib/bbac266 35817399

[B51] Tang X. Luo J. Shen C. Lai Z. (2021). Multi-view multichannel attention graph convolutional network for mirna–disease association prediction. Brief. Bioinform. 22, bbab174. 10.1093/bib/bbab174 33963829

[B52] Wang C.-C. Han C.-D. Zhao Q. Chen X. (2021a). Circular rnas and complex diseases: From experimental results to computational models. Brief. Bioinform. 22, bbab286. 10.1093/bib/bbab286 34329377 PMC8575014

[B53] Wang L. Wong L. Li Z. Huang Y. Su X. Zhao B. (2022a). A machine learning framework based on multi-source feature fusion for circrna-disease association prediction. Brief. Bioinform. 23, bbac388. 10.1093/bib/bbac388 36070867

[B54] Wang L. Yan X. You Z.-H. Zhou X. Li H.-Y. Huang Y.-A. (2021b). Sganrda: Semi-supervised generative adversarial networks for predicting circrna–disease associations. Brief. Bioinform. 22, bbab028. 10.1093/bib/bbab028 33734296

[B55] Wang L. You Z.-H. Huang D.-S. Li J.-Q. (2021c). Mgrcda: Metagraph recommendation method for predicting circrna-disease association. IEEE Trans. Cybern. 2021, 1–9. 10.1109/TCYB.2021.3090756 34236991

[B56] Wang S. Wang Y. Cheng H. Zhang Q. Fu C. He C. (2022b). The networks of noncoding rnas and their direct molecular targets in myocardial infarction. Int. J. Biol. Sci. 18, 3194–3208. 10.7150/ijbs.69671 35637964 PMC9134914

[B57] Wang W. Dai Q. Li F. Xiong Y. Wei D.-Q. (2021d). Mlcdforest: Multi-label classification with deep forest in disease prediction for long non-coding rnas. Brief. Bioinform. 22, bbaa104. 10.1093/bib/bbaa104 32520339

[B58] Wang W. Guan X. Khan M. T. Xiong Y. Wei D.-Q. (2020). Lmi-dforest: A deep forest model towards the prediction of lncrna-mirna interactions. Comput. Biol. Chem. 89, 107406. 10.1016/j.compbiolchem.2020.107406 33120126

[B59] Wapinski O. Chang H. Y. (2011). Long noncoding rnas and human disease. Trends Cell. Biol. 21, 354–361. 10.1016/j.tcb.2011.04.001 21550244

[B60] Wu H. Liang Q. Zhang W. Zou Q. Hesham A. E.-L. Liu B. (2022a). ilncda-ltr: Identification of lncrna-disease associations by learning to rank. Comput. Biol. Med. 2022, 105605. 10.1016/j.compbiomed.2022.105605 35594681

[B61] Wu H. Wu Y. Jiang Y. Zhou B. Zhou H. Chen Z. (2022b). schicstackl: a stacking ensemble learning-based method for single-cell hi-c classification using cell embedding. Brief. Bioinform. 23, bbab396. 10.1093/bib/bbab396 34553746

[B62] Xiao Q. Fu Y. Yang Y. Dai J. Luo J. (2021). Nsl2cd: Identifying potential circrna–disease associations based on network embedding and subspace learning. Brief. Bioinform. 22, bbab177. 10.1093/bib/bbab177 33954582

[B63] Xu H. Hu X. Yan X. Zhong W. Yin D. Gai Y. (2022). Exploring noncoding rnas in thyroid cancer using a graph convolutional network approach. Comput. Biol. Med. 145, 105447. 10.1016/j.compbiomed.2022.105447 35430557

[B64] Xu J. Cai L. Liao B. Zhu W. Yang J. (2020). Cmf-impute: An accurate imputation tool for single-cell rna-seq data. Bioinformatics 36, 3139–3147. 10.1093/bioinformatics/btaa109 32073612

[B65] Yang J. Ju J. Guo L. Ji B. Shi S. Yang Z. (2022). Prediction of her2-positive breast cancer recurrence and metastasis risk from histopathological images and clinical information via multimodal deep learning. Comput. Struct. Biotechnol. J. 20, 333–342. 10.1016/j.csbj.2021.12.028 35035786 PMC8733169

[B66] Ye F. Zhang G. Chen H. Yu C. Yang L. Fu Y. (2022). Construction of the axolotl cell landscape using combinatorial hybridization sequencing at single-cell resolution. Nat. Commun. 13, 4228. 10.1038/s41467-022-31879-z 35869072 PMC9307617

[B67] Yu A.-M. Jian C. Allan H. Y. Tu M.-J. (2019). Rna therapy: Are we using the right molecules? Pharmacol. Ther. 196, 91–104. 10.1016/j.pharmthera.2018.11.011 30521885 PMC6450780

[B68] Yu N. Liu Z.-P. Gao R. (2022). Predicting multiple types of microrna-disease associations based on tensor factorization and label propagation. Comput. Biol. Med. 146, 105558. 10.1016/j.compbiomed.2022.105558 35525071

[B69] Zhang L. Yang P. Feng H. Zhao Q. Liu H. (2021a). Using network distance analysis to predict lncrna–mirna interactions. Interdiscip. Sci. 13, 535–545. 10.1007/s12539-021-00458-z 34232474

[B70] Zhang S. He X. Zhang R. Deng W. (2021b). Lncr2metasta: A manually curated database for experimentally supported lncrnas during various cancer metastatic events. Brief. Bioinform. 22, bbaa178. 10.1093/bib/bbaa178 32766766

[B71] Zhang T. Chen L. Li R. Liu N. Huang X. Wong G. (2022a). Piwi-interacting rnas in human diseases: Databases and computational models. Brief. Bioinform. 23, bbac217. 10.1093/bib/bbac217 35667080

[B72] Zhang W. Qu Q. Zhang Y. Wang W. (2018). The linear neighborhood propagation method for predicting long non-coding rna–protein interactions. Neurocomputing 273, 526–534. 10.1016/j.neucom.2017.07.065

[B73] Zhang Y. Wang D. Peng M. Tang L. Ouyang J. Xiong F. (2021c). Single-cell rna sequencing in cancer research. J. Exp. Clin. Cancer Res. 40, 81–17. 10.1186/s13046-021-01874-1 33648534 PMC7919320

[B74] Zhang Z. Cui F. Cao C. Wang Q. Zou Q. (2022b). Single-cell rna analysis reveals the potential risk of organ-specific cell types vulnerable to sars-cov-2 infections. Comput. Biol. Med. 140, 105092. 10.1016/j.compbiomed.2021.105092 PMC8628631 34864302

[B75] Zhang Z. Wang Z.-X. Chen Y.-X. Wu H.-X. Yin L. Zhao Q. (2022c). Integrated analysis of single-cell and bulk rna sequencing data reveals a pan-cancer stemness signature predicting immunotherapy response. Genome Med. 14, 45–18. 10.1186/s13073-022-01050-w 35488273 PMC9052621

[B76] Zhao X. Zhao X. Yin M. (2022). Heterogeneous graph attention network based on meta-paths for lncrna–disease association prediction. Brief. Bioinform. 23, bbab407. 10.1093/bib/bbab407 34585231

[B77] Zhou G. Jiang N. Zhang W. Guo S. Xin G. (2021a). Biomarker identification in membranous nephropathy using a long non-coding rna-mediated competitive endogenous rna network. Interdiscip. Sci. 13, 615–623. 10.1007/s12539-021-00466-z 34472046

[B78] Zhou L. Wang Z. Tian X. Peng L. (2021b). Lpi-deepgbdt: A multiple-layer deep framework based on gradient boosting decision trees for lncrna–protein interaction identification. BMC Bioinforma. 22, 479. 10.1186/s12859-021-04399-8 PMC8489074 34607567

[B79] Zhou P. Wang S. Li T. Nie Q. (2021c). Dissecting transition cells from single-cell transcriptome data through multiscale stochastic dynamics. Nat. Commun. 12, 5609–5615. 10.1038/s41467-021-25548-w 34556644 PMC8460805

